# Radioactive Iodine Therapy and Glucose Tolerance

**DOI:** 10.22074/cellj.2016.4251

**Published:** 2017-02-22

**Authors:** Roghaieh Samadi, Babak Shafiei, Fereidoun Azizi, Asghar Ghasemi

**Affiliations:** 1 Endocrine Physiology Research Center, Research Institute for Endocrine Sciences, Shahid Beheshti University of Medical Sciences, Tehran, Iran; 2 Department of Nuclear Medicine, Taleghani Hospital, Shahid Beheshti University of Medical Sciences, Tehran, Iran; 3 Endocrine Research Center, Research Institute for Endocrine Sciences, Shahid Beheshti University of Medical Sciences, Tehran, Iran

**Keywords:** Radioactive, Iodine, Glucose Tolerance, Pancreas, Sodium-Iodide Symporter

## Abstract

Radioactive iodine therapy is commonly used as an adjuvant therapy in follicular and papillary thyroid carcinoma (PTC) and in the treatment of Graves’ disease (GD). The basis of this therapy is the accumulation of radioactive iodine by the sodium-iodide symporter (NIS) in the thyroid gland. Expression of NIS by extrathyroidal tissues such as islets of pancreas has been reported. Radioactive iodine uptake by pancreatic beta-cells can potentially damage these cells. In this study, we discuss the possible associations between radioactive iodine and glucose intolerance. Overall, radioactive iodine uptake by the pancreas may damage beta-cells and predispose patients to glucose intolerance or type 2 diabetes, particularly in patients exposed to radioactive iodine therapy following total thyroidectomy. Further studies are needed to clarify and confirm this association.

## Introduction

Radioactive iodine therapy is commonly used as an adjuvant therapy in follicular and papillary thyroid carcinoma (PTC) and in the treatment of Graves’ disease (GD) (1). Iodine-131 is a radioisotope of iodine that emits beta and gamma radiation. Production of reactive oxygen species (ROS) by beta radiation destroys living cells. Radioactive iodine is taken up and concentrated in thyroid follicular cells by the membrane transporter, i.e., the sodium-iodide symporter (NIS) where it subsequently produces ionizing radiation that ablates the thyroid remnant and eliminates any suspected micrometastases (2,4). Although NIS is mostly expressed in thyroid tissue, it is also found in other tissues that capture radioactive iodine, including the stomach, salivary glands, thymus, nasal mucosa, lacrimal glands, and lactating breasts (5,6). Radioactive iodine therapy could, therefore, exert side effects in these tissues (7,9). It has been reported that the pancreas expresses NIS (10,11). A few studies report that radioactive iodine can impair glucose metabolism (12,13). Radioactive iodine can potentially damage pancreatic beta-cells and predispose patients to glucose intolerance or even type 2 diabetes. The aim of this study is to discuss possible associations that may exist between radioactive iodine and glucose intolerance. 

### Hyperthyroidism

is one of the most common endocrine disorders (14). GD is the most frequent cause of hyperthyroidism and most often seen in women, aged 20 to 40 years (15). The incidence of GD in adult women has been reported to be 4.6 per 1000 during a 10 year follow-up in the Nurses’ Health Study II from the United States (16). GD is an autoimmune disease caused by a sharp rise of thyroid stimulating immunoglobulin (TSI), which mimics the action of thyroid stimulating hormone (TSH) and increases production of T3 and T4 by the thyroid gland (17). GD is associated with increased levels of serum anti-thyroperoxidase antibody (anti-TPOAb). Levels are higher among in women than men and increase with age (18). 

### Thyroid cancer

Thyroid cancer is the most frequent cancer of the endocrine system (19), which occurs mostly in women aged <45 years with an age-adjusted incidence that is three times higher in women than men (19,20). In women, thyroid cancer is the fifth most prevalent cancer (21). Although the incidence of thyroid cancer is increasing rapidly in men, its higher incidence in women impacts overall trends. The estimated mortality in women is 1.17-fold higher than in men (21,22). Thyroid follicular epithelial-derived carcinomas are divided into PTC, follicular thyroid carcinoma (FTC), and anaplastic thyroid carcinoma (ATC) (23,24). PTC and FTC are the most common forms. Together, they are considered as differentiated thyroid cancer (DTC) (25). PTC accounts for approximately 80% of all thyroid cancers, whereas FTC comprises 10-15%, and ATC accounts <2% of all thyroid cancers (14,23,24). The mortality rate related to follicular carcinoma is >2-fold higher than papillary carcinoma (26). 

### Radioactive iodine therapy in thyroid cancer and hyperthyroidism

#### Treatment of thyroid cancer and hyperthyroidism 

The first line of treatment for thyroid carcinoma is surgery, except for certain cases of ATC. Total or near-total thyroidectomy is recommended for the majority of PTC and FTC patients (14). External-beam radiation is also considered for older patients (>45 years) with extensive PTC in whom complete surgical excision is impossible and in patients whose tumors do not concentrate radioactive iodine (14,27). After thyroidectomy, radioactive iodine therapy is used in patients with distant organ metastases, incomplete tumor resection, or complete tumor resection with a high risk of recurrence and mortality (28). Since TSH controls the growth of thyroid tumor cells, levothyroxine therapy is also useful to inhibit TSH secretion after surgery (29). Differentiated thyroid tumors are often treatable, but ATC is the most aggressive thyroid cancer and gives poor response to treatment (23). 

Antithyroid drugs (i.e., thionamides), total or partial thyroidectomy, and radioactive iodine therapy are the current treatments available for hyperthyroidism (3,30,31). Surgery is rarely performed because of its potential risks that include laryngeal nerve injury, damage to the parathyroid glands, and permanent hypothyroidism (32,33). Patients are usually unwilling to take antithyroid drugs (methimazole, propylthiouracil, and carbimazole) for treatment of hyperthyroidism because they must be taken for a long time. Many patients experience relapse when the antithyroid drugs are withdrawn after 12-18 months of treatment (34,36). Radioactive iodine was introduced as a treatment for hyperthyroidism in 1941 because of its safety, low cost, and rapid effects (1). The relapse rate of hyperthyroidism after treatment with antithyroid drugs is 53%, whereas for surgery it is 14, and 8% for radioactive iodine therapy (37). It has been reported that the use of antithyroid drugs two weeks before or after radioactive iodine therapy resulted in a significant reduction in cure rates in patients who received 5 mCi radioactive iodine, but not 10 mCi (38). 

#### Radioactive iodine

The best-known radioisotopes of iodine are iodine-123, iodine-124, iodine-125, and iodine-131. Iodine-123 (half-life 13 hours) is used for diagnostic imaging, whereas iodine-124 (half-life 4.18 days) is mostly used for positron emission tomography (PET scanning) (39). Iodine-125 (half-life 59 days) is used in imaging and radioimmunoassays as well as for labeling proteins. Iodine-131, a radioisotope of iodine with a physical half-life of 8.1 days (2,39), is converted to stable X-131 (a stable isotope of xenon) and emits beta and gamma radiation during the decay process (39). Compared with beta particles, gamma radiation has a low energy of 0.364 megaelectronvolt (MeV) and can be used diagnostically. Beta particles have maximum energy of 0.61 MeV and travel a distance of about 2 mm in tissues; high linear energy transfer (LET) of beta particles is considered in radioactive iodine therapy (2,40). Iodine is vital for the proper functioning of the thyroid gland to produce T4 and T3 (41). Thyroid cells cannot distinguish between radioactive and non-radioactive iodine (iodine-127); in addition its metabolism, biodistribution and excretion are the same as for iodine-127 (42,43). 

#### Sodium-iodide symporter in the thyroid gland

The NIS is a membrane glycoprotein that belongs to the solute carrier family 5A (SLC5A). All members of this protein family are sodium- dependent transporters (44). Human NIS (h-NIS) is a 643-amino acid glycoprotein with 13 membrane-spanning domains, an intracellular carboxyl-terminus, and an extracellular amino- terminus localized at the basolateral plasma membrane of thyrocytes (45,46). The mature NIS protein has a molecular weight of 80-90 kDa with three asparagine-linked glycosylation sites; although glycosylation does not seem to be required for activity or correct targeting of the protein to the plasma membrane, it plays a role in protein stabilization and folding (47). TSH is the primary regulator of NIS expression and upregulates NIS mRNA and protein expression (48). NIH has a longer half-life (5 days) in the presence of TSH than in its absence (3 days) (49). Iodine uptake is an essential first step in the synthesis of thyroid hormones. Thyroid hormones play fundamental roles in the development, metabolism, and growth (50,51). NIS cotransports two sodium ions along with one iodide and transmembrane sodium gradient that provides the driving force for iodide uptake (52,53). Under physiological conditions, the thyroid gland concentrates iodine by a factor of 20-40 times compared to plasma (14). 

#### Sodium-iodide symporter and radioactive iodine therapy

**Fig.1:**
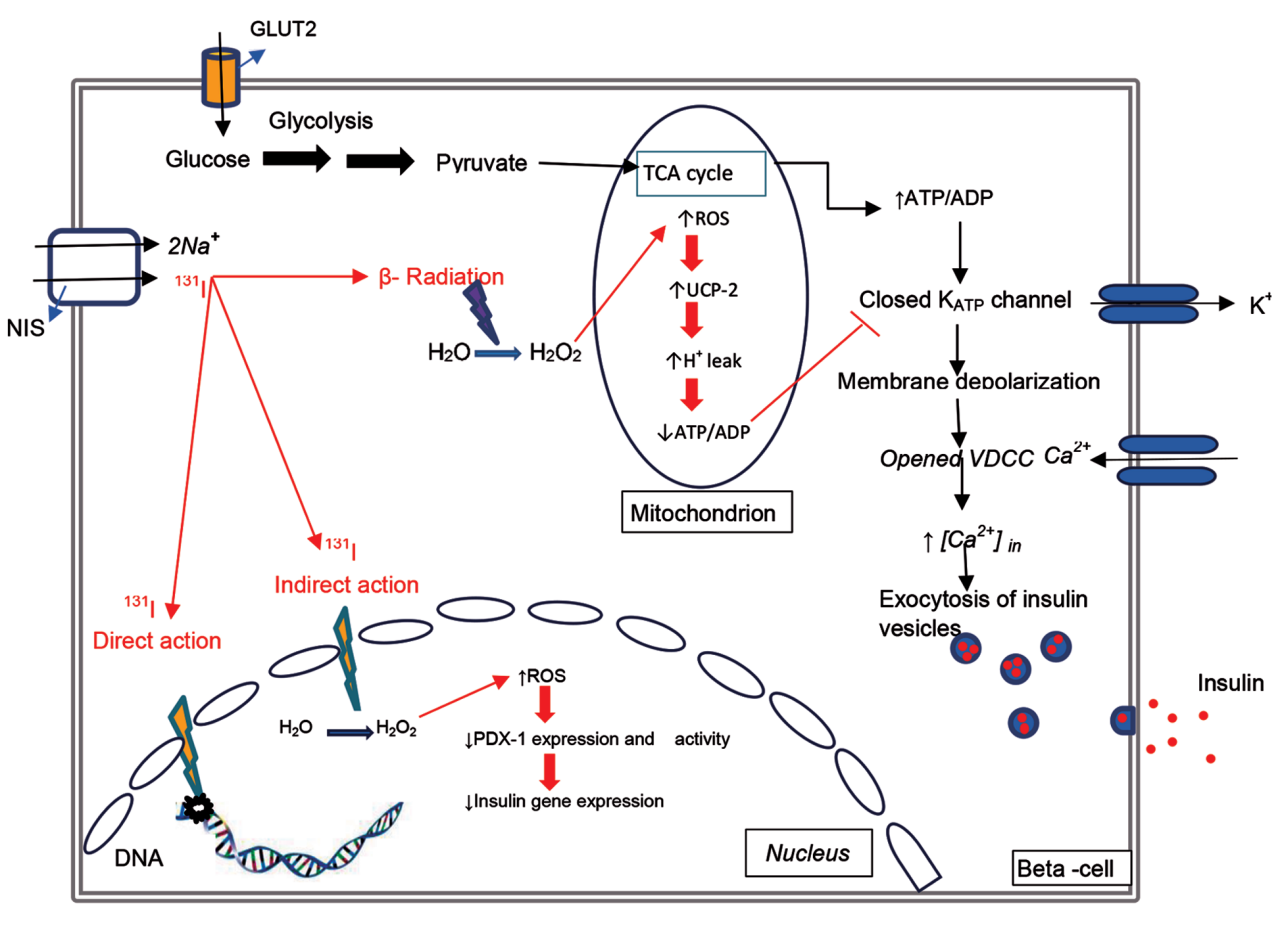
Glucose enters beta-cells through glucose transporter 2 (GLUT2) and converts to pyruvate in the glycolysis pathway. Pyruvate enters the mitochondrion to be metabolized further and produce ATP. An increased ATP/ADP ratio is necessary for insulin secretion. I-131 (radioactive iodine) enters beta-cells via the sodium-iodide symporter (NIS) and produces reactive oxygen species (ROS). Increased ROS in beta-cells activates uncoupling protein-2 (UCP-2) that decreases the ATP/ADP ratio, leading to suppressed glucose-stimulated insulin secretion (GSIS). Directly, beta-particles interact with essential molecules and disrupt DNA, while indirectly, beta-particles produce ROS by partial reduction of oxygen (O2) and interaction with water molecules. Increased ROS in the nucleus decreases pancreatic duodenal homeobox-1 (PDX-1) expression and activity, and decreases insulin gene expression. TCA; Tricarboxylic acid and VDCC; Voltage-dependent calcium channel.

The ability of the thyroid gland to concentrate radioactive iodine provides the basis for therapeutic management of benign thyroid diseases as well as for thyroid cancer (42). Following administration of radioactive iodine, a major amount is concentrated in the thyroid gland by NIS. Ionizing radiation (beta- particles) has a short range and can cause cell damage and death (2,54,55). As shown in Figure 1, ionizing radiation causes cell injury by direct and indirect actions. In the direct action, beta-particles interact with essential molecules and disrupt DNA (56), while indirectly beta-particles produce ROS by partial reduction of oxygen (O_2_) and interaction with water molecules (57), after which ROS reacts with cellular macromolecules, nucleic acids, proteins, and lipids (54,58). 

#### Radioactive iodine in treatment of hyperthy- roidism and thyroid cancer

The ability of the thyroid gland to accumulate radioactive iodine is important in the diagnosis and treatment of thyroid disorders (4,59). Increased thyroidal NIS mRNA and protein expression in benign thyroid diseases such as GD (3-4 fold compared to the normal thyroid) allows effective therapy with radioactive iodine (60). Although radioactive iodine therapy has been stablished for treating GD, the approach to dosing however remains controversial due to different goals for treatment (control of hyperthyroidism vs. avoidance of hypothyroidism) (61). The radioactive iodine dose to treat hyperthyroidism is 5-15 mCi and patients given a single dose of 10 mCi have higher cure rates than those given 5 mCi. However, no significant difference has been reported in the cure rate of hyperthyroidism for patients who received 10 mCi, compared with 15 mCi (38,62).

Postoperative radioactive iodine therapy is 187 used for both FTC and PTC, which originate from the follicular cells and can accumulate radioactive iodine (25); however, thyroid tumor cells accumulate less radioactive iodine than normal thyroid cells (63,64). Postoperative radioactive iodine is used to ablate a thyroid remnant, remove suspected micrometastases, and decrease thyroid cancer mortality (4). Compared to GD, higher dosages of radioactive iodine (25 to >200 mCi) are required for treatment of thyroid carcinoma (28,59). It has been shown that low doses of radioactive iodine (29-50 mCi) are as effective as high doses (51 to 200 mCi) in controlling tumor recurrence (7 vs. 9%). In addition, the recurrence rate following radioactive iodine therapy is one-third that following thyroid hormone therapy per se (26). 

**Table 1:** Expression of the sodium-iodide symporter (NIS) in extrathyroidal human tissues


Tissues	Protein expression	mRNA expression	
	Immunohistochemistry	Northern blot	RT-PCR

Pancreas	+ (11, 65), – (66)		+ (10)
Mammary glands			+ (11, 67)
Salivary glands	+ (10, 66)	– (67)	+ (11, 67)
Stomach	+ (66)	– (67)	+ (67)
Intestinal fat		– (67)	
Lacrimal gland	+ (10)		
Gastric mucosa	+ (10)		+ (11)
Ovarian tissue	– (66)		+ (11), – (67)
Rectum	+ (66)		
Colon	+ (10)	– (11, 67)	
Skin	– (66)		– (67)
Appendix	– (66)		
Testis		– (11)	+ (11)
Adrenal glands		– (11)	
Kidneys	– (66)		
Prostate		+ (11)	
Spleen	– (66)		
Esophagus	– (66)		


Numbers in parentheses are references. +; NIS present, –; NIS not present, and RT-PCR; Reverse transcription-polymerase chain reaction.

### Radioactive iodine therapy and pancreas dysfunction

#### Distribution of sodium-iodide symporter in extrathyroidal tissues

NIS expression is not restricted only to the thyroid, rather, it has been found in extrathyroidal tissues (Table 1). A high concordance rate in the results between immunohistochemistry and reverse transcription-polymerase chain reaction (RT-PCR) has been reported for NIS expression in extrathyroidal tissues. Results of Northern blot analyses, however, were negative in all tissues despite positive results obtained by immunohistochemistry and RT-PCR. It has been reported that the RT-PCR assay was more sensitive than Northern blot for assay of mRNA expression (68).

#### Side effects of radioactive iodine therapy

Because of expression of NIS in extrathyroidal tissues, side effects of radioactive iodine therapy are expected. High doses of radioactive iodine (25-500 mCi) have early and late complications. Early complications include dry eye (69), pregnancy and ovarian complications (fetal thyroid ablation, birth defects and growth retardation) (70), gastritis, nausea and vomiting (6), taste changes (8), bone marrow suppression (7), and hypospermia (71). Late complications include leukemia and secondary cancers (72, 73), pulmonary fibrosis (70), and chronic dry eye (74). Compared to antithyroid drugs (75), after radioactive iodine therapy, antigens (thyroglobulin and thyroperoxidase) are released into circulation and activate T cells (76). This could explain why this therapy may be associated with secondary immunological disturbances (76, 77) that increase the risk for worsening of Graves’ ophthalmopathy.

#### Sodium-iodide symporter expression in the pancreas

Although the transfer of iodine in extrathyroidal tissues is TSH-independent, some studies indicate that iodine organification also occurs in tissues other than the thyroid (78). Localization of radioactive iodine in the lungs, liver, muscles, intestinal mucosa, pancreas, spleen, and thymus in swine tissues has been reported (79). There are conflicting reports of NIS expression in the pancreas (10,65,66). Spitzweg et al. (10,11) have reported NIS immunoreactivity in ductal cells, exocrine parenchymal cells, and islets of Langerhans in the human pancreas with a high degree of positive staining in islets of Langerhans and weaker staining in duct cells and parenchymal exocrine cells (11). Wapnir et al. (65) have also reported that pancreatic islets and exocrine pancreatic cells are immunoreactive and express NIS. Mitsuma et al. (80) reported that islets of Langerhans in rats expressed NIS, which was detectable by immunohistochemistry staining. 

### Radioactive iodine causes beta-cell dysfunction

Beta-cells are especially sensitive to ROS, because of their low expression of anti-oxidant enzymes that include catalase and glutathione peroxidase compared to other tissues such as the liver (81). Radioactive iodine enters cells and causes ROS-induced damage (57); in addition, ionizing radiation transmitted from adjacent organs can also contribute to this damage (82). H_2_O_2_ is non-polar and can diffuse freely across membranes over a very long distance (83); it can thus enter the mitochondria and nuclear membrane. 

### Radioactive iodine therapy and insulin secretion

In beta-cells, radioactive iodine-derived ROS activates uncoupling protein-2 (UCP-2) (84), which mediates mitochondrial proton leak and decreases ATP production. Increased ATP/ADP ratio is necessary for insulin secretion, therefore ROS-derived from radioactive iodine, in particular H_2_O_2_, can lead to suppression of glucose stimulated insulin secretion (GSIS) (85). Islet ATP levels are higher in UCP-2-deficient mice and lead to increased GSIS, thus UCP-2 hampers insulin secretion (86). Consistent with this observation, overexpression of UCP-2 in beta-cells has been reported to impair GSIS (87).

Pancreatic duodenal homeobox-1 (PDX-1), a member of the homeobox transcription factor family, is an insulin promoter activator (88) expressed in the pancreas and duodenum. PDX-1 plays a crucial role in maintenance of endocrine and exocrine function of the mature pancreas by regulating many important beta-cell genes, including insulin, GLUT2, and glucokinase (89, 90). PDX-1 also contributes to pancreas development (91, 92) and the regeneration and differentiation of beta-cells (93, 94). Glucose toxicity and accumulation of ROS reduce DNA-binding to PDX-1 in beta-cells (95, 96). Oxidative stress causes PDX-1 translocation from the nucleus to the cytoplasm of pancreatic beta-cells thus inhibiting PDX-1 nuclear localization and DNA binding through the activation of the c-Jun N-terminal kinase (JNK) pathway (97, 98). It has been shown that oxidative stress activates the JNK pathway causing reduction of insulin gene expression. Rat islets are protected from oxidative stress when the JNK pathway is suppressed (99).

#### Radioactive iodine therapy and glucose tolerance

Accumulation of radioactive iodine in the pancreas could potentially lead to impaired glucose tolerance and diabetes mellitus. Few studies have investigated the effects of radioactive iodine on pancreatic function. Hallengren et al. (100) studied the effects of radioactive therapy in hyperthyroid patients. They reported no significant difference between HbA1C before and six months after radioactive iodine therapy. However, the doses of radioactive iodine used were not mentioned. Kiani et al. (13) reported that radioactive iodine treatment (5-13 mCi) had no adverse effects on glucose tolerance and insulin resistance in Graves’ patients (n=73) compared to those treated with methimazole (n=59). In addition, median 2-hour blood glucose and serum insulin levels were comparable, and no significant difference existed in the medians of 2-hour blood and fasting glucose levels between those who received <10 mCi radioactive iodine and those that received >10 mCi. Two limitations of this study were: i. Medians of 2-hour blood glucose and serum insulin levels were not measured before treatment and ii. It would have been better if thyroid cancer patients that received high doses of radioactive iodine were enrolled in this study. Accumulation of radioactive iodine in the pancreas is inhibited in the presence of the thyroid gland; thyroid-cancer patients who have undergone thyroidectomies and are exposed to radioactive iodine may therefore be good candidates for evaluation of the radioactive iodine effects on glucose tolerance (101). 

Although details are not available, Durmaz et al. 

(12) reported significantly greater fasting glucose levels of hyperthyroid patients treated with a single therapeutic dose of radioactive iodine (8- 15 mCi) compared to pretreatment levels. The results were inconsistent with those by Kiani et al. (13) and Hallengren et al. (100). Considering the limited data on the effect of radioactive iodine on pancreas function, more studies are needed to demonstrate the possible effects of radioactive iodine on glucose tolerance. 

## Conclusion

Radioactive iodine is used for treatment of GD and as an adjuvant in the treatment of thyroid cancer. Radioactive iodine is concentrated in the thyroid gland by NIS and can cause cell damage and death in the thyroid; other tissues such as islets of Langerhans can also uptake radioactive iodine. 

Radioactive iodine enters beta-cells by NIS and produces ROS, which damage beta-cells. ROS also activates UCP-2, which suppresses GSIS. In addition to the ROS-mediated indirect pathway, radioactive iodine can also directly damage DNA. In conclusion, very few studies have been conducted on the possible associations between radioactive iodine and pancreas function. Despite limitations of the available studies, radioactive iodine uptake by the pancreas may damage beta- cells and predispose patients to glucose intolerance or even type 2 diabetes, particularly those exposed to radioactive iodine therapy following total thyroidectomy. Nevertheless, more studies are needed to determine and confirm possible effects of radioactive iodine on glucose tolerance. 
